# P05 Phenotypic detection of MRSA in crustacean seafood, Lagos, Nigeria

**DOI:** 10.1093/jacamr/dlaf230.012

**Published:** 2025-12-04

**Authors:** Akaso Mujeeb, O E Ojo, Michael Ajibare

**Affiliations:** Federal University of Agriculture, Abeokuta, Ogun State, Nigeria; Federal University of Agriculture, Abeokuta, Ogun State, Nigeria; Federal University of Agriculture, Abeokuta, Ogun State, Nigeria

## Abstract

**Background:**

The increasing emergence of antimicrobial-resistant pathogens including MRSA poses a significant threat to food safety and public health. The unhygienic handling practices combined with environmental contamination and antibiotic misuse in Lagos Nigeria's coastal communities make crustacean seafood a potential reservoir for MRSA.

**Methods:**

A total of 71 crustacean seafood samples were randomly collected from three major seafood markets across Lagos State .The Baird Parker Agar served as a medium for *Staphylococcus aureus* isolation followed by Gram staining and biochemical tests to confirm the results. The Kirby–Bauer disc diffusion method on Mueller–Hinton agar followed CLSI guidelines to test presumptive isolates for antibiotic susceptibility. The phenotypic marker tests using cefoxitin (30 μg) and oxacillin (5 μg) identified MRSA strains. The presumptive *S. aureus* isolates from 71 samples reached 60 (84.5%) and the phenotypic resistance tests showed 22 (36.67%) and 60 (100%) isolates were resistant to cefoxitin and oxacillin respectively. Six out of 21 processed isolates showed resistance to cefoxitin and all 21 processed isolates showed resistance to oxacillin. The 39 fresh isolates showed 16 cefoxitin-resistant strains but all 39 samples displayed complete resistance to oxacillin.

**Results:**

The antibiotic susceptibility profile revealed that each of Augmentin (100%), meropenem (100%), ceftriaxone (100%) and linezolid (100%) showed high resistance while ciprofloxacin exhibited relatively lower resistance.

**Conclusions:**

The research demonstrates that MRSA exists in crustacean seafood products sold in Lagos which creates potential health risks for both consumers and handlers. The high level of MDR indicates that antibiotics have been used either excessively or improperly in aquatic environments and post-harvest handling processes. The recommended measures to control resistant strain spread include regular surveillance and proper seafood handling practices together with controlled antimicrobial use in aquaculture operations. This study provides essential local findings about MRSA in seafood which supports the requirement for One Health-based AMR monitoring in Nigeria.

